# Spatial patterns of recent US summertime heat trends: Implications for heat sensitivity and health adaptations

**DOI:** 10.1088/2515-7620/ab7abb

**Published:** 2020-03-11

**Authors:** Keith R Spangler, Gregory A Wellenius

**Affiliations:** 1 Department of Earth, Environmental, and Planetary Sciences, Brown University, Providence, RI, United States of America; 2 Department of Epidemiology, Brown University School of Public Health, Providence, RI, United States of America; 3 Institute at Brown for Environment and Society, Brown University, Providence, RI, United States of America; 4 Department of Environmental Health, Boston University School of Public Health, Boston, MA, United States of America; 5 Current address: Department of Environmental Health, Boston University School of Public Health, Boston, MA, United States of America

**Keywords:** climate change, extreme heat, human health, heat index

## Abstract

Heat is known to cause illness and death not only at extreme temperatures, but also at moderate levels. Although substantial research has shown how summer time temperature distributions have changed over recent decades in the United States, less is known about how the heat index—a potentially more health-applicable metric of heat—has similarly evolved over this period. Moreover, the extent to which these distributional changes have overlapped with indicators of social vulnerability has not been established, despite the applicability of co-varying climatic and sociodemographic characteristics to heat-related health adaptations. Presented here is an analysis of trends in the median, 95th percentile, and ‘warm-tail spread’ (i.e., intra-seasonal range between the upper extreme and median) of warm-season (May-September) maximum heat index between 1979and 2018 across the conterminous US. Using40 years of data from the North American Regional Reanalysis dataset, it is shown that most of the US has experienced statistically significant positive trends in summertime heat, and that both the magnitude of trends and the shape of the frequency distributions of these measures vary regionally. Comparisons with data from the Social Vulnerability Index show that the most socially vulnerable counties appear to be warming faster than the least vulnerable, but that opposite patterns hold for trends in warm-tail spread. These findings may be applicable to further studies on climate change, heat adaptations, and environmental justice in the US.

## 1. Introduction

The relationship between moderate-to-extreme heat and risk of mortality is well established [1], as is the observation that global mean surface temperatures have increased significantly over the last century [2]. However, there is substantial spatial heterogeneity not only in these warming trends, but also in the underlying population characteristics that affect susceptibility to heat health effects [3]. In the context of adapting to the epidemiologic impacts of climate change, there is consequently interest in identifying the locations that are currently experiencing the greatest rates of moderate-to-extreme summertime warming and to determine the extent to which such warming intersects with population-scale indicators of social vulnerability.

Since heat-related mortality risks increase starting at relatively modest temperatures[4], the overall health burden on the ‘warm tail’ of a summertime temperature distribution is characterized both by high-frequency, low-impact days at moderate levels and by low-frequency, high-impact days at the upper extreme. However, previous assessments of health-relevant climatic trends tend to focus on the latter and quantify changes in terms of days exceeding an *a priori* threshold, such as record-breaking high ambient temperatures[5, 6], or changes to the frequency of ‘heat-wave days’ [7–9] or other extreme climatic indices[10]. Since these analyses exclude a large portion of health-applicable heat, trends in the changes to the full distribution of moderate-to-extreme values are preferable for understanding how populations have experienced climate change and its health effects.

The role of variability changes emerges as an important additional consideration, not only because within-season temperature variability itself has been associated with increased mortality risks [11, 12], but also because changes to the mean and extreme values of a distribution are interrelated and affected by alterations to the shape of the distribution [13]. While there is debate as to whether *global* temperatures have experienced increased variability over the last century [14–16], there appears to be regional heterogeneity [17]. In the US, Rhines *et al* [18] found substantial spatial differences by season in the direction and magnitude of the spread of upper and lower extremes of ambient temperatures. Gross *et al* [19] expanded this globally and reached similar conclusions about the seasonally and regionally dependent nature on changes to temperature extremes relative to changes in the mean. Looking specifically at summertime temperatures, McKinnon *et al* [20] also found substantial regional heterogeneity in ambient temperature trends and suggested that distributional changes in most places were largely explained by shifts, though changes to variability also played a role in some places.

It remains unclear, however, whether these distributional changes also apply to the *heat index*, a measure of apparent temperature that approximates human thermal comfort by incorporating both ambient temperature and relative humidity [21], and which is widely used both in epidemiologic research (e.g., Kim *et al* [22], Bell *et al* [23], Michelozzi *et al* [24], and Wellenius *et al* [25]) and in operational contexts for the issuance of heat warnings [26]. Although Grotjahn and Huynh [27] found increases in summertime maximum heat index for much of the US in recent decades, they did not consider distributional changes, which may differ from those of temperature yet are of interest for similar reasons.

The extent to which any of these indicators of climatic change could affect heat mortality, however, depends on the extent to which the changes overlap with sociodemographic factors that alter the heat-mortality relationship, such as age and pre-existing medical conditions [3]. These factors, which affect the ability to prepare for, respond to, and recover from hazards that can cause harm, are reflective of *social vulnerability* [28, 29]. Despite the importance of this intersection to climate change adaptations, the literature appears to lack an assessment of the co-occurrence of heat index warming trends and vulnerability.

To address the knowledge gaps identified above, the analysis presented here will answer two questions: (1) To what extent is there regional variability in the distributional changes of warm-season (May-September, MJJAS) maximum heat index (*HI*_*max*_) in the contiguous United States (CONUS) and (2) Are the most socially vulnerable counties experiencing distributional changes in *HI*_*max*_ that are different from the least-vulnerable counties? We hypothesized that CONUS observed significant increases in the median and upper extremes of *HI*_*max*_ with strong regional heterogeneity both in the magnitude of warming and in the sign of variability changes. We further hypothesized that these spatially differential distributions resulted in significant differences in heat trends between the most- and least-vulnerable counties.

We tested these hypotheses by calculating recent trends (1979–2018) in the distribution of warm-season *HI*_*max*_ throughout CONUS and assessing how these changes intersect with county-level indicators of social vulnerability. We calculated rates of change in the annual warm-season median and 95th percentile of *HI*_*max*_ to respectively characterize changes in the central tendency and upper extreme of heat, and we assessed trends in the annual seasonal difference between the 95th percentile and median values of *HI*_*max*_ to identify systematic changes in intra-seasonal warm-tail variability. We then compared these rates by National Climate Assessment (NCA) regions to identify regional heterogeneity in trends. Finally, we pooled the climatic changes at the county scale by levels of the Social Vulnerability Index [30] and compared by top and bottom deciles to identify differences.

## 2. Methods

### 2.1. Datasets

#### 2.1.1. Climate data

**Figure 1.**
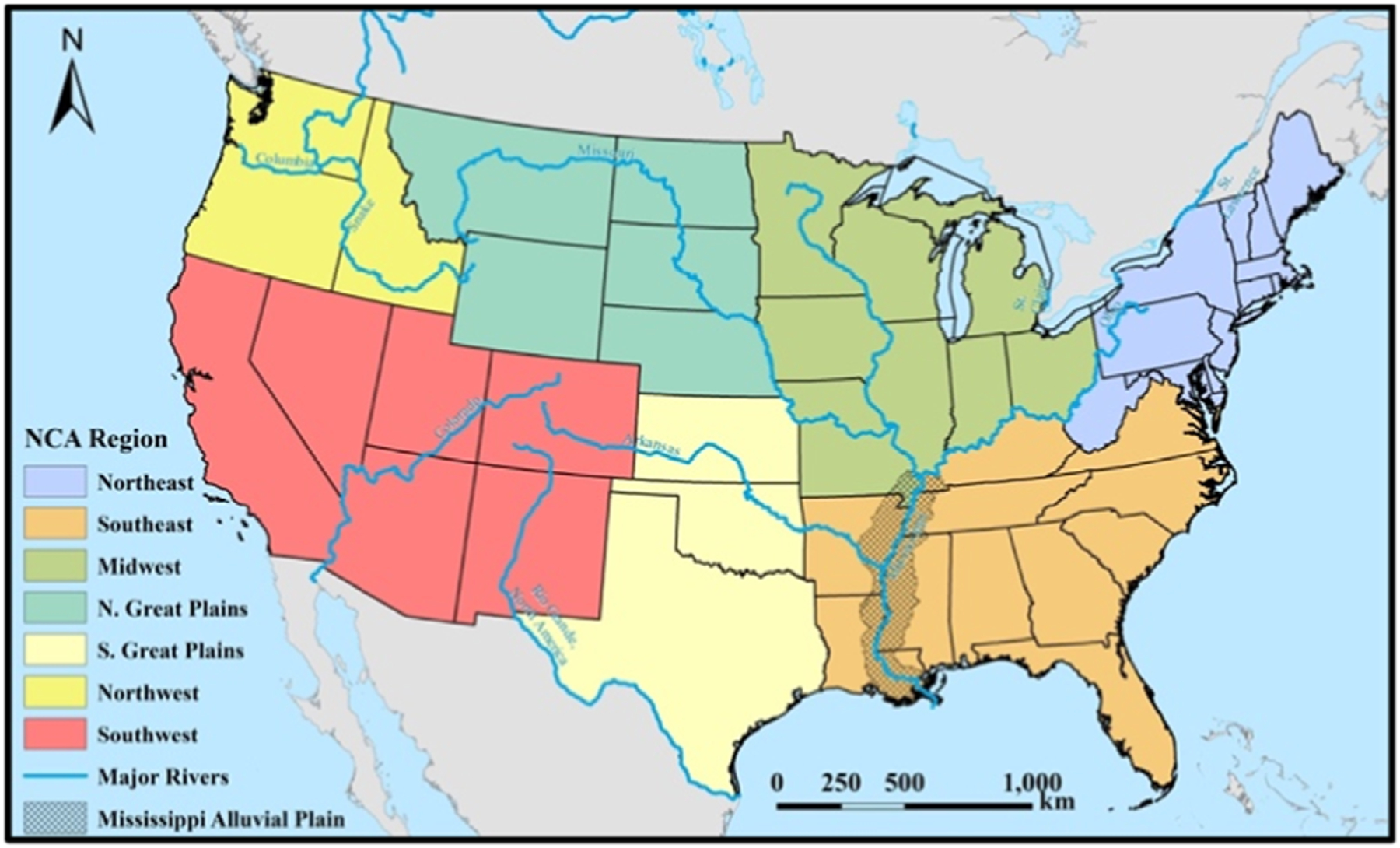
Map of the contiguous United States (CONUS) with major rivers, divided into National Climate Assessment (NCA) regions. Cross-hatching indicates the Mississippi Alluvial Plain in the Southeast (data from the U.S. Environmental Protection Agency [33]). Background mapping and river data provided by ArcWorld and ArcWorld Supplement from Esri®.

The North American Regional Reanalysis (NARR) product [31, 32] was used in the assessment of observed historical trends of heat index. The NARR data are available for all of North America at three-hourly increments from 1 January 1979 through present, with a horizontal grid spacing of approximately 32 kilometers. The data were subset to the months of May-September (‘warm season’) for all available years (1979–2018) and to the area of interest, CONUS (figure 1).

#### 2.1.2. Social vulnerability data

We used the Social Vulnerability Index (SVI) from the Centers for Disease Control and Prevention [30] to measure relative differences in sociodemographic indicators of vulnerability. This dataset provides percentile rankings of census tracts and counties across the United States based on population proportions of characteristics believed to be correlated with negative health outcomes from exposure to hazards and catastrophes [34]. An SVI of 0 reflects the location with the lowest relative vulnerability in the US and a value of 100 reflects the highest relative vulnerability. The overall SVI contains all of the variables that can be further divided into four themes: Theme 1 is socioeconomic status, including variables on poverty, unemployment, per-capita income, and lack of higher education; Theme 2 is on household characteristics, including age (elderly or children), disability, and single parenthood; Theme 3 is on race, ethnicity, and language, including variables on the population proportion of racial or ethnic minorities and proportion with limited English-language proficiency; and Theme 4 is on housing and transportation, including variables on multi-unit structures, mobile homes, overcrowding, lack of access to personal vehicle, and group quarters. See Flanagan *et al* [34] for a full description. We used the county-level SVI values to align more closely with the 32-km resolution of the climate data and the 2016 product to most closely reflect present-day social vulnerability.

### 2.2. Calculating heat metrics

We calculated daily maximum heat index (*HI*_*max*_) from the eight observations available for each day between 1 May and 30 September (MJJAS) for 1979–2018. For each of the eight three-hourly observations, we calculated *HI*_*max*_ using the weathermetrics R package, which applies the algorithm employed by the US National Weather Service, described elsewhere [21]. Two-meter (i.e., near surface) ambient air temperature (T) and relative humidity (RH) served as inputs to these calculations. The seasonal (MJJAS) median and 95th percentile values (approximately the seventh-hottest day of the warm season) of *HI*_*max*_ were derived from these daily measures for each pixel in each of the 40 years in the dataset.

To quantify the rate of change in recent historical observations of heat, we fit ordinary least-squares (OLS) regression models for each individual pixel on the seasonal (MJJAS) median (50th percentile) *HI*_*max*_ values of each year, and then repeated the process separately for the seasonal 95th percentiles. As a way to measure trends in the intra-seasonal warm-tail variability, additional regressions were fit on annual trends in the difference between the 95th and 50th percentiles within each year (*HI*_*95-med*_); a positive trend would indicate greater variability (i.e., more spread between an upper extreme and median), a negative trend would reflect decreasing variability (i.e., less spread between the median and extreme), and a lack of trend would indicate either the absence of climatic change or commensurate changes in the median and extreme (i.e., a shift of the distribution with no change in shape). The OLS models resulted in regression coefficients (i.e., slope) and standard errors for each pixel across CONUS that indicated the average change over time in the seasonal median, 95th percentile, and intra-annual difference between them (95th—median) for maximum heat index. For ease of interpretation, trends are reported in units of °C per decade.

Since slopes of best-fit lines are particularly influenced by values at the beginning and end of the time series, we varied the start and end years by five years each, for a total of 25 permutations of time periods. The shortest period was 32 years (1983–2014) and the longest was 40 years (1979–2018). We then used standard meta-analytic methods [35] to pool the results from the 25 separate regression models in each pixel using the meta for R package [36]. Pixels with meta-analytic p-values < 0.05 were considered statistically significant. Hereafter, ‘1979–2018’ refers to the meta-analysis of the 25 models spanning this period.

### 2.3. Assessing county-level differences

**Figure 2.**
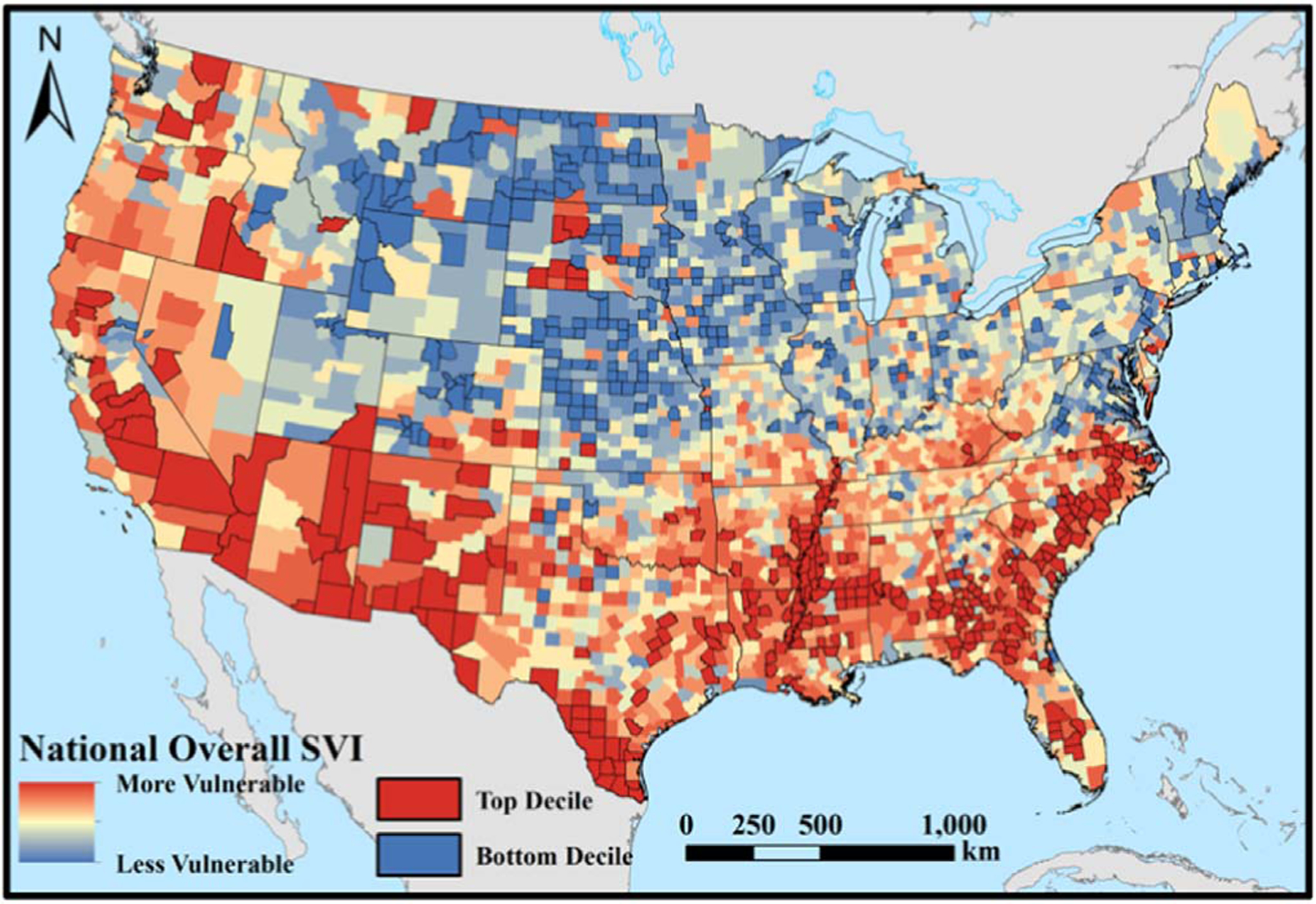
Map showing the distribution of county-level overall 2016 Social Vulnerability Index (SVI) values. The top- and bottom-decile counties are outlined and in red and blue, respectively. Background mapping provided by ArcWorld and ArcWorld Supplement from Esri®.

We estimated county-level trends in the warm-season heat metrics by assigning the regression coefficient from the pixel located at the 2010 population centroid for each county, as provided by the US Census Bureau [37]. We assigned county-level trends in this way primarily to maximize the applicability to human exposures: while most counties are relatively small compared to the resolution of the reanalysis data, many counties in the western US are large and sparsely populated; identifying the trends occurring at the location of the population centroid may therefore provide a better estimate of the potential changes actually experienced by human populations. To determine whether the most-vulnerable counties have experienced different rates of change in the heat metrics compared to the least-vulnerable counties, we performed two-tailed Welch’s *t*-tests [38] on the regression coefficients for each heat variable in the top- and bottom-decile counties (SVI ≥ 90th and SVI ≤ 10th percentile nationally) (figure 2). The Welch’s *t*-test determines whether the difference in means between two groups is statistically significant and, unlike the traditional Student’s *t*-test, does not assume equal variance or sample sizes between the comparison groups [39]. Finally, for the purpose of creating a preliminary visualization of the intersection between SVI and trends in *HI*_*max*_, we used min-max standardization to transform each value onto a consistent scale from 0–1 and then computed averages. Values closer to one would indicate counties with both relatively high social vulnerability and rates of warming and vice versa for values closer to zero.

## 3. Results

### 3.1. Warm-season heat trends

**Figure 3.**
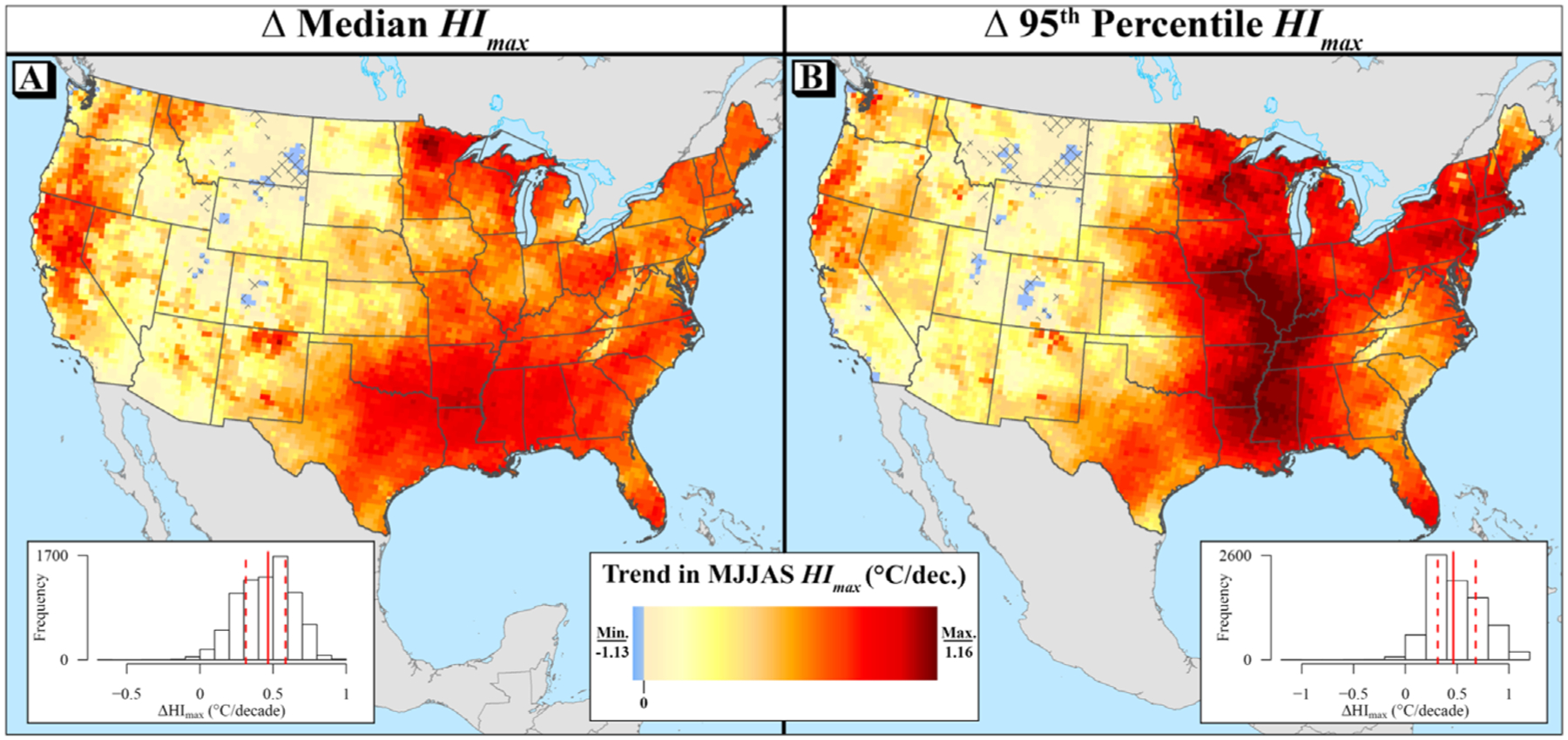
Linear trends (°C/decade) in May-September (MJJAS; warm season) daily maximum heat index (*HI*_*max*_) for the median (a) and 95th percentile (b) for 1979–2018. Cross-hatching indicates that the trend is not statistically significant (meta-analytic p-value ≥ 0.05). Solid red lines on histograms represent the median value for the contiguous US (CONUS), while the dashed red lines indicate the first and third quartiles. Background mapping provided by ArcWorld and ArcWorld Supplement from Esri®.

Virtually all of the conterminous US has observed statistically significant warming trends in both the median and 95th percentiles of annual warm-season *HI*_*max*_ between 1979–2018 (figure 3). Although the sign of the trend is mostly homogenous throughout CONUS, there is substantial spatial variability in the degree of warming between regions (figure 3). The northern Great Plains show the least amount of warming, with either no statistically significant trend or a very small positive or negative trend. By contrast, parts of northern Minnesota and the south-central US (Louisiana, Mississippi, and Arkansas) around the Mississippi Alluvial Plain (see again: figure 1) have amongst the fastest rates of change in both of the heat metrics assessed. The remaining parts of CONUS have somewhat differing magnitudes in the trends, depending on the variable assessed; for example, the northeastern US has relatively faster warming in the 95th percentile of *HI*_*max*_ than the median and vice versa for the coastal southeastern US.

**Figure 4.**
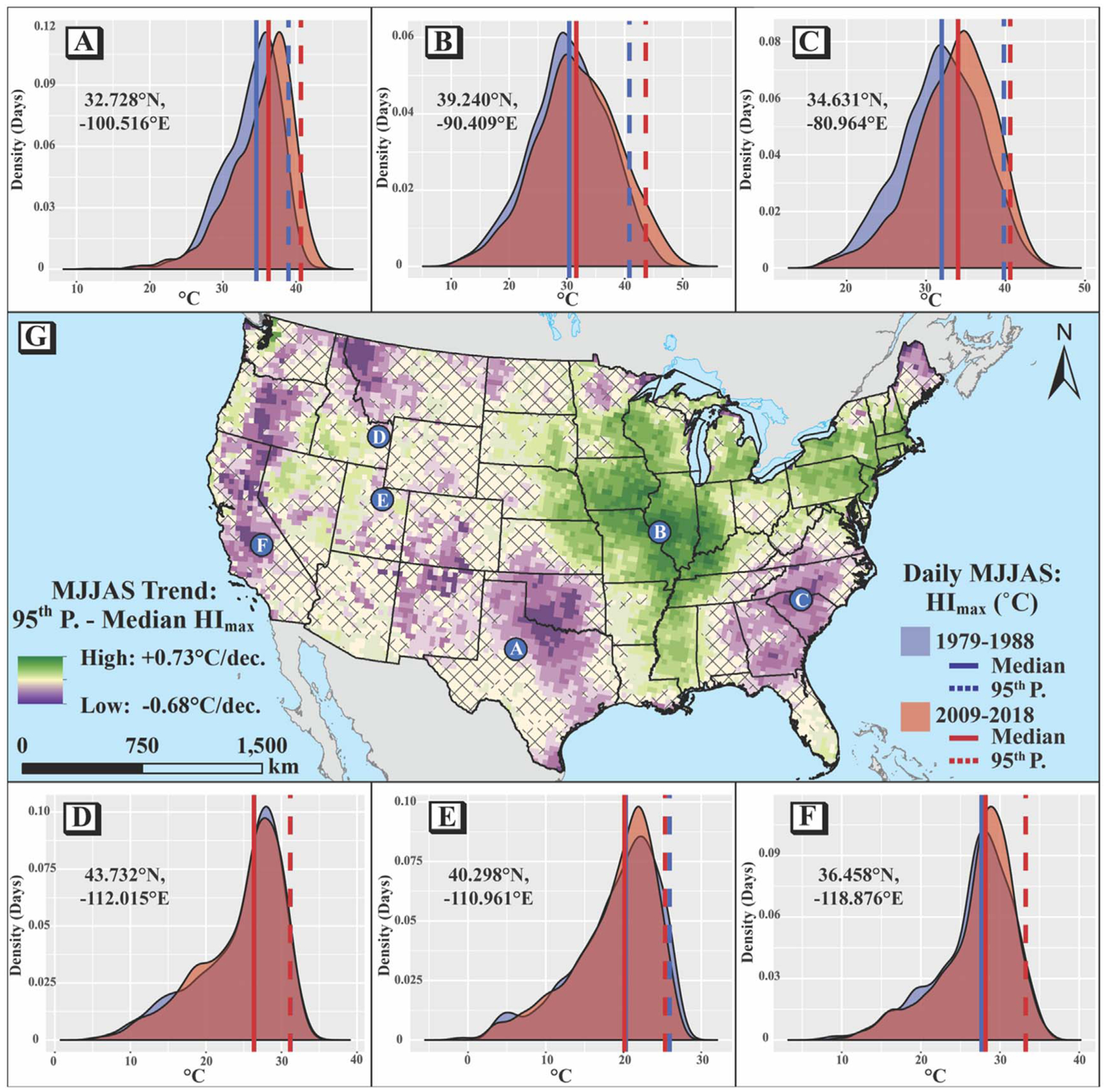
Trends in annual difference between the 95th percentile and median heat index (*HI*_*95-med*_) for 1979–2018 (g). Purple indicates places where the median and 95th percentile values have gotten closer together (decreasing within-season ‘warm-tail’ variability), green indicates where they have diverged (increasing spread), and cross-hatching indicates that the trend is not statistically significant (meta-analytic p-value ≥ 0.05). Density plots show examples at selected pixels of how these distributional changes can manifest, with red curves representing *HI*_*max*_ values for 2009–2018 and blue curves representing the earlier decade of 1979–1988 (a–f). Solid lines on the density plots are median values for that decade and the dashed lines reflect the 95th percentiles. Background mapping provided by ArcWorld and ArcWorld Supplement from Esri®.

Differences in the magnitude of warming trends in the median and extreme values suggest that climatological changes do not always manifest as simple rightward shifts in the distribution of maximum warm-season heat index at more-localized scales. Although many parts of CONUS have observed commensurate changes in the median and 95th percentile *HI*_*max*_, other areas show either convergent or divergent trends (figure 4(g)): places with no trend in the difference between annual 95th percentile and median *HI*_*max*_ (indicated by cross-hatching) experienced no statistically significant change in the spread of warm-tail MJJAS *HI*_*max*_, while places with positive values (green) saw increasing spread, and places with negative values (purple) saw decreasing spread. The density plots in figure 4(a–f) compare the frequency of all daily MJJAS *HI*_*max*_ values for the first decade of the period (1979–1988, blue curves) to the final decade (2009–2018, red curves) at select pixels as clarifying illustrations; places with no significant trend in *HI*_*95-med*_ have approximately equal widths between the median (solid vertical line) and 95th percentile (dashed vertical line) for the earlier and later decades (blue and red, respectively), places with significant positive trends in the *HI*_*95-med*_ have increasing widths, and places with significant decreasing trends have decreasing widths.

**Figure 5.**
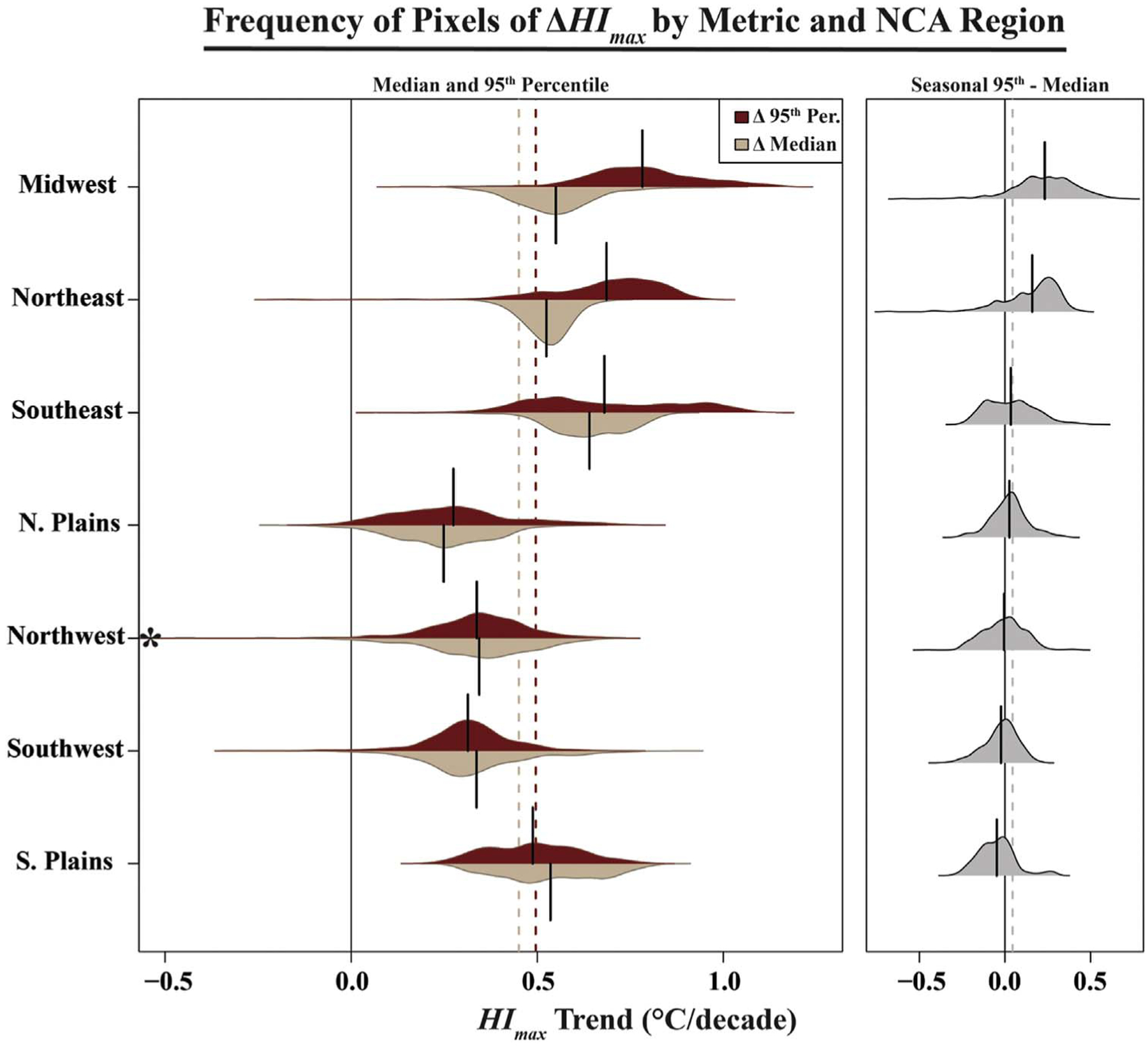
Density plots showing frequency of decadal trends (1979–2018) in warm-season (MJJAS) maximum heat index (*HI*_*max*_) across CONUS by seasonal median and 95th percentile (left plots) and warm-tail spread (seasonal 95th percentile minus median; right plots) across the seven National Climate Assessment (NCA) regions. Solid lines on individual plots indicate mean values and dashed lines across plots indicate the CONUS average. Plots reflect frequency of NARR pixels containing at least 25% land. Asterisk (*)indicates one pixel in the Northwest that goes beyond the scale of the x-axis (values of this outlier: −0.67°C/decade [median] and −1.13°C/decade [95^th^ percentile]).

There appears to be an east-west component in the trends of *HI*_*95-med*_: most of the eastern US has increasing trends (tendency toward greater spread of *HI*_*max*_), while there is more heterogeneity of values west of the Mississippi River. A notable exception is the coastal Southeast, which has a fairly strong negative trend. Although there are some localized instances of statistically significant decreases in either median or 95th percentile of *HI*_*max*_ (see again figure 3), the vast majority of CONUS experienced increases in one or both of these metrics. Therefore, the trends in *HI*_*95-med*_ suggest that, in general, the Southern Great Plains, coastal Southeast, and parts of the west coast have experienced faster increases in the median *HI*_*max*_ than in 95th percentiles, while the Northeast, Midwest, and Mississippi Alluvial Plain have experienced faster increases in 95th percentiles than medians of *HI*_*max*_ (figure 5).

### 3.2. Heat trends by social vulnerability index

**Figure 6.**
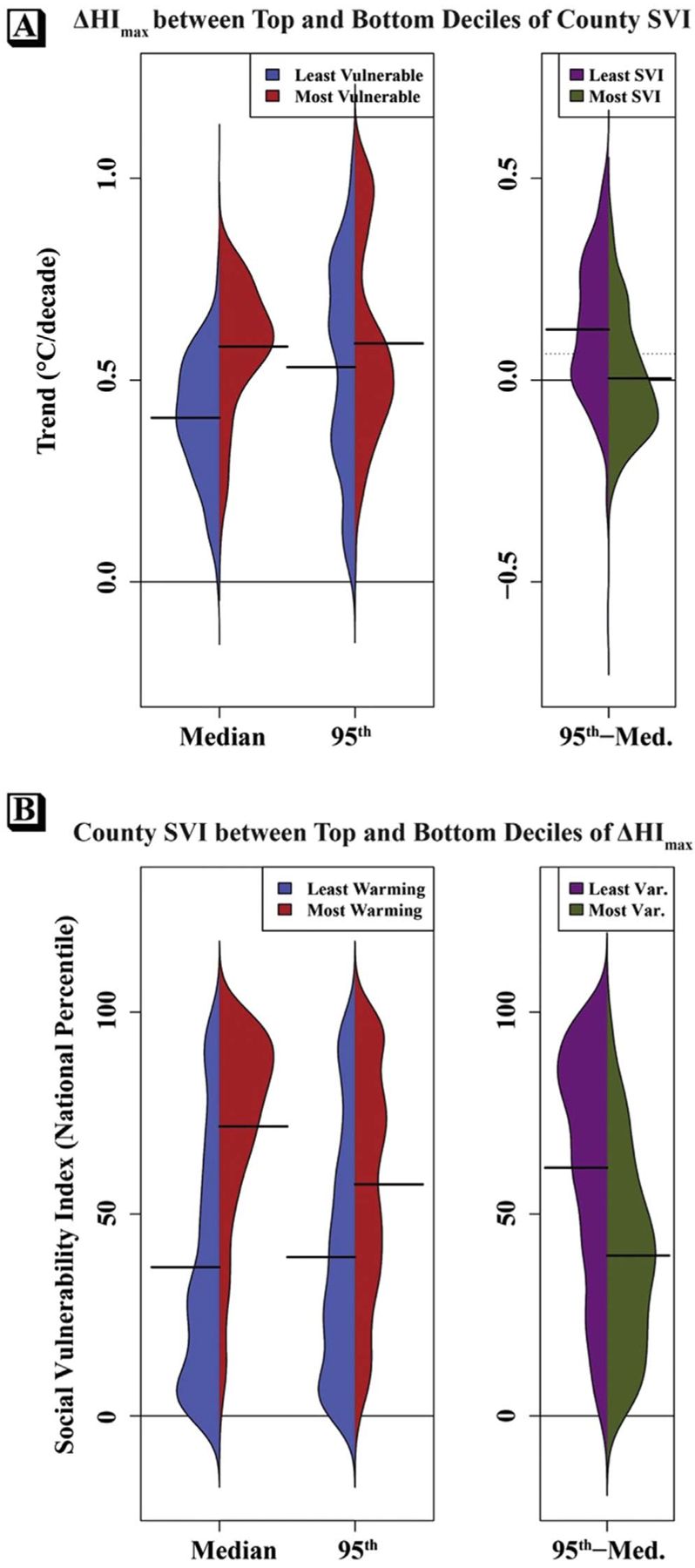
Plots showing the frequency of county-level trends (1979–2018) in MJJAS median and 95th percentile of *HI*_*max*_ (left) and in the intra-seasonal spread (95th–median, right) between the top and bottom deciles of county SVI (a), and plots showing the frequency of county-level SVI values within the top-10% fastest warming and bottom-10% slowest warming counties (b).

Comparisons of climatic heat trends between the top and bottom deciles of SVI by county show that, on average, the most-vulnerable counties (those with SVI values in the top 10% nationally) are warming statistically significantly faster than the least-vulnerable counties (those with SVI values in the bottom 10%) for both median and 95th percentile of *HI*_*max*_, with the former difference approximately triple the latter (figure 6(a)). The t-tests show that the differences in means for the trends between the top and bottom deciles of county SVI are 0.18°C/decade (95% CI [0.15, 0.20]) for median *HI*_*max*_ and 0.06°C/decade (95% CI [0.02, 0.10]) for 95th percentile *HI*_*max*_. However, the most-vulnerable counties, on average, have experienced no change in warm-tail spread (0.004°C/decade) while the least-vulnerable counties have experienced an increase (0.126 °C/decade); the t-test difference in these means is −0.12 °C/decade (95% CI [−0.15, −0.10]).

The relationships hold when considering differences in SVI between the most- and least-warming counties (figure 6(b)): the difference in mean SVI values between the most- and least-warming counties is 34.8 percentage points (95% CI [30.6, 39.0]) for median *HI*_*max*_ and 18.0 percentage points (95% CI [13.5, 22.5]) for 95th percentile *HI*_*max*_. By contrast, the mean SVI in counties with the greatest increase in warm-tail spread is 39.7 percentage points, compared to 61.5 percentage points in the least-variable decile; the t-test difference in these means is −21.8 percentage points (95% CI [−25.8, −17.7]).

**Figure 7.**
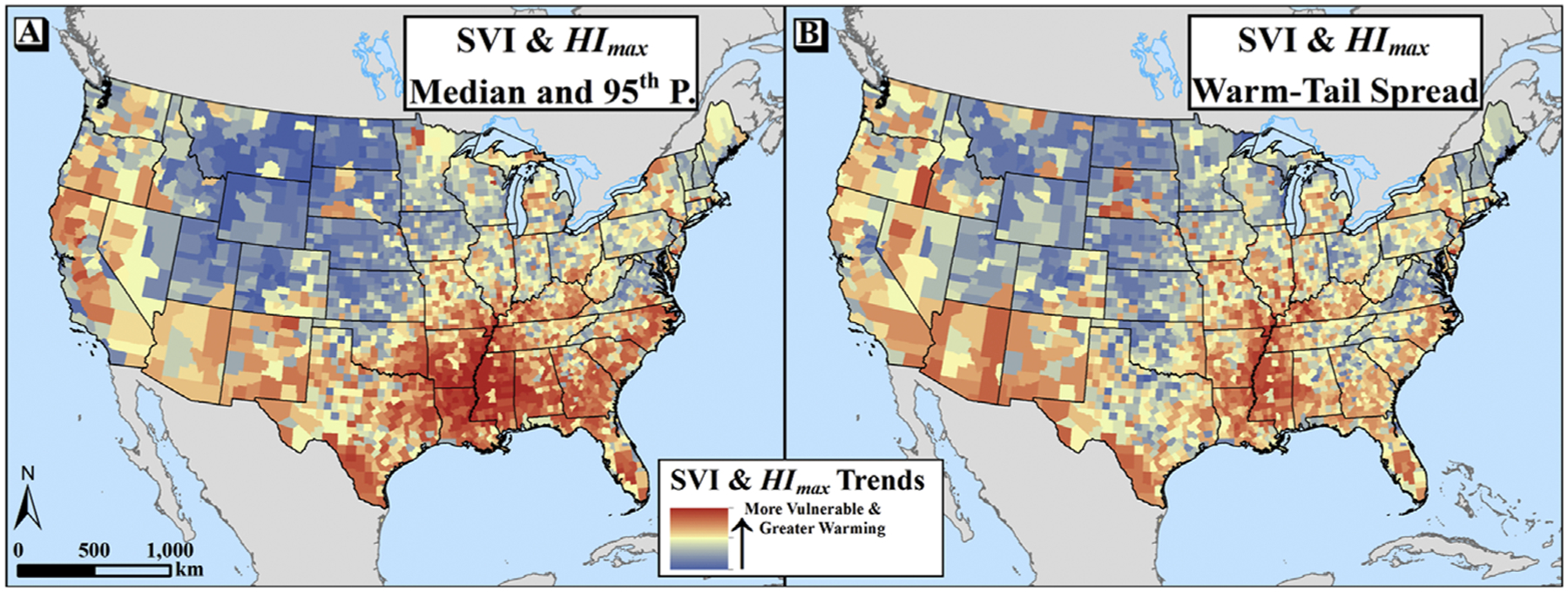
Maps showing the spatial intersection of the Social Vulnerability Index and min-max standardized values of recent trends in *HI*_*max*_. In (a), the SVI is averaged with the mean of the standardized trends in the median and 95th percentile, while in (b) the SVI is averaged with the standardized value of the warm-tail spread (measured by the change in the seasonal 95th percentile minus median). Background mapping provided by ArcWorld and ArcWorld Supplement from Esri®.

Substantial spatial heterogeneity in the intersection of SVI and *HI*_*max*_ trends can be seen both for changes in the median and 95th percentile (figure 7(a)) and in the warm-tail spread, albeit to a lesser degree (figure 7(b)). In both cases, the Southeast appears to have the greatest coincidence of SVI and *HI*_*max*_ trends, and the Mississippi Alluvial Plain region appears to have particularly high values across all metrics.

## 4. Discussion

In this paper, we presented an analysis of recent trends in warm-season (MJJAS) daily maximum heat index by median, 95th percentile, and intra-annual warm-tail spread (the within-season difference between the 95th and 50th percentiles) for all of the contiguous United States between 1979–2018. While these data showed that virtually all of CONUS has experienced statistically significant *HI*_*max*_ warming trends over this period, regional heterogeneity in magnitudes was apparent: on average, rates of change in median *HI*_*max*_ were highest in the Southeast and lowest in the Northern Great Plains, while trends in 95th percentile *HI*_*max*_ were highest in the Midwest and lowest in the Northwest. Strong spatial patterns were also observed in the within-season warm-tail variability: The Northeast and Midwest had particularly high increases in spread, while the Southern Great Plains, coastal Southeast, and parts of the west coast had decreases. Finally, on average, the most-vulnerable counties were shown to be warming faster than the least-vulnerable counties (particularly for median *HI*_*max*_), but the latter has experienced an increase in warm-tail spread, while the former has not. These findings are novel both for showing distributional changes in the *heat index* (rather than ambient temperature) and for showing a potential relationship between recent summertime warming trends and indicators of social vulnerability. In the discussion that follows, we contextualize the trend findings in the broader literature on moderate-to-extreme heat changes and explains the significance of these trends—in tandem with the co-occurrence with SVI—to heat-health effects and climate adaptation.

Comparing the findings presented here to existing studies is challenging, since conclusions drawn from climatic trend analyses can differ substantially based on the years assessed, variables of interest, geographic extent, months considered, dataset employed, and methodologies applied [40]; nonetheless, some qualitative comparisons are made here. In a recent paper, Grotjahn and Huynh [27] assessed trends in mean and maximum heat index for the June-August (JJA) months of three time periods over the 20th Century and compared these trends between gridded observational datasets and several reanalysis products. For their most-recent period (1979–2011), the observational datasets showed warming across most of CONUS for JJA *HI*_*mean*_ and all but one of the reanalysis products showed warming over the majority of CONUS for *HI*_*max*_ (although two of the reanalyses showed large areas of non-significant cooling trends in the central US). Although the reanalyses had different patterns and magnitudes, they mostly all agreed on warming in the eastern US, which the authors note is ‘intensified’ relative to changes in maximum ambient temperature, in some places reversing a cooling trend. These findings are consistent with our results for heat-index warming, which appears particularly amplified in the moist eastern US. Although the literature appears to lack an analogous assessment of a continuous measure of *extreme* heat index (e.g., seasonal 95th percentile), several studies have found regionally varying increases in heat waves using criteria based on the heat index (e.g., Lyon and Barnston [7] and Smith *et al* [9]). Although the arbitrary nature of heat wave definitions makes comparisons difficult, the finding in Smith *et al* [9] that the southeastern US appears to have amongst the highest rates of increase from 1979–2011 across most of the heat wave definitions considered is qualitatively consistent with our finding showing particularly strong warming in both the median and 95th percentile of *HI*_*max*_ around the Mississippi Alluvial Plain.

To visualize *distributional* changes in warm-season heat, we additionally quantified trends in the within-season difference between the extreme and median values of *HI*_*max*_. This assessment complements the broader literature on ambient temperature distributional changes, which also finds regional differences in the signs of variability and other statistical moments. Rhines *et al* [18] calculated trends from 1979–2014 in the 5th and 95th percentiles, as well as the spread between them, by season across CONUS for *T*_min_ and *T*_max_ using an observational dataset. During JJA, they found decreasing *T*_max_ trends (i.e., reduced spread) for the coastal Southeast, Southwest, and the northern Great Plains, and they found increasing trends (i.e., expanded spread) for the south-central US and part of the Northwest. McKinnon *et al* [20] also found substantial regional heterogeneity in station-observed ambient *T*_max_ trends (1980–2015) in the US and suggested that the distributional changes in most places were largely explained by shifts, though decreasing variability was also shown for all of CONUS except for the south-central US, which showed an increase. Increased skewness and kurtosis were also shown for the Northeast, Midwest, and parts of the western US. Finally, Gross *et al* [19] assessed global ‘excess change’ between the mean and 98.5th percentile of temperature anomalies by season since the mid-20th Century. For maximum temperature, their analysis of the observational data showed extreme maximum temperatures in JJA increasing faster than the mean in the south-central, northern-Midwest, and part of the northeastern US, while the west coast and southeast largely had greater increases in the mean relative to the upper extreme. This pattern of differential warming qualitatively concurs with our findings here that showed increasing warm-tail *HI*_*max*_ variability in the Midwest and Northeast but decreasing variability for the coastal Southeast and west coast. Signs are opposite, however, for Texas and the nearby Mississippi Alluvial Plain.

Additional research is needed to explain the differences between our findings and some of the trends in the temperature-based analyses found elsewhere. Particularly noteworthy is the lack of cooling that appears in the trends of weather-station based assessments of summertime *T*_max_ in some studies (e.g., Rhines *et al* [18] and McKinnon *et al* [20]). This could be due partly to inhomogeneities in weather station observations over this period, such as those introduced by the transition from liquid-in-glass thermometers to thermistor-based instruments in the 1980s that prompted an average cooling of 0.4°C to daily maximum temperatures [41]. In contrast to the widespread cooling seen in the *unadjusted* station-based assessments, NOAA Climate Division (NCD) data—which account for a variety of inhomogeneities [42]—presented in Grotjahn and Huynh [27] show that only one climate division in the Midwest had a cooling trend of JJA *T*_max_ between 1979–2011, though a larger area in the Midwest showed no trend. The use of NARR data, which is a reanalysis product and thus assimilates a variety of observational data [31], may be less sensitive to this potential bias. While there is some debate regarding limitations in the use of reanalysis data for trend analyses [43, 44], the principle concern of inhomogeneities from the assimilation of new data sources is mostly allayed for the post-satellite era after 1979 [45]. Moreover, others have demonstrated that NARR agrees well with temperature trends of annual mean temperatures across CONUS [46, 47]. Nonetheless, our findings we presented here should be interpreted with the caveat that other datasets, methodologies, and period of interest could produce different results [40].

Of course, differences between changes in heat index and ambient temperature are attributable, in part, to atmospheric moisture content. Specifically, the stronger trends in median and extreme heat index observed in the eastern United States are likely partly indicative of the underlying humidity climatology because: (1) the eastern US has much more atmospheric moisture than the western US on average [48], meaning that a particular increase in temperature would yield a greater increase in the heat index, given the nonlinearity of the heat-index algorithm [21]; and (2) others have observed that the central and eastern US experienced increasing trends in relative humidity over the last quarter of the 20th Century [49]. Less certain, however, are the drivers of the regional heterogeneity in the changing *spreads* of the warm-tail distributions. While a comprehensive assessment of the myriad geophysical causes of our findings is outside the scope of this analysis, it should be noted that the role of soil moisture in land-atmosphere interactions and climate feedbacks, particularly for extreme heat, has been extensively documented in the literature (see Seneviratne *et al* [50] for a review). The influence of anomalously dry soils, which can affect the overall distribution of ambient temperatures by reducing the cooling effect of latent heat of evaporation and increasing the warming effect of sensible heat fluxes [51], has been shown to play a particularly dominant role in extreme heat events (e.g., Brabson *et al* [52] and Whan *et al* [53]). Given that soil moisture affects both ambient temperature and relative humidity [54], it follows that a change in soil moisture could also play a role in changing heat index distributions. However, the extent to which such changes in warm-season soil moisture content are regionally covariable with the *HI*_*max*_ distributional changes observed in this paper remains an open question.

Regardless of the causes, the spatial heterogeneity observed in these heat metrics is applicable to adaptation broadly and to heat-related health interventions specifically. Others have noted that distributional changes to the extremes versus the mean present different challenges to adaptation [55]. In particular, increases in variability can push conditions outside the ‘coping range’ [56], which is compounded by co-occurring warming. Those places that have simultaneously experienced increases in the median, 95th percentile, and spread (particularly the Midwest, Mississippi Alluvial Plain, and the Northeast) constitute an example of this because the changing summertime heat patterns are not only warmer on average, but also more extreme and more variable, making it more difficult for populations to adapt to these changes. We posit that such communities would need to build the greatest amount of *adaptive capacity* (the ability to take actions that reduce harm from climate change [57]), to minimize the resultant heat-related health effects. *Acclimatization*, the physiological processes that enable the human body to adapt to changing temperatures on both sub-seasonal and interannual timescales[58, 59], is one mechanism by which this could be achieved. However, temperature-related mortality may be greatest when it is ‘unusual’ (i.e., anomalous given the time of year or the location), suggesting that increases in variability may be of greater public health concern than just an increase in average temperatures [60], perhaps because it could stymie acclimatization. Therefore, regions trending toward greater warm-tail variability may face greater challenges to adaptation, while those with reduced warm-tail variability could potentially be better equipped to adapt to changes in heat.

Critically important to this assessment of adaptation, however, is the underlying social vulnerability of the populations experiencing the changes. We showed here that, across CONUS, the most-vulnerable decile of counties is warming faster than the least-vulnerable decile, especially for median *HI*_*max*_. Given that populations with higher social vulnerability are less able to prepare for, and recover from, natural hazards [28, 29], it follows that the combination of enhanced warming and greater vulnerability may have a compounding effect on climatic *sensitivity*, i.e., ‘the degree to which a system or species is affected, either adversely or beneficially, by climate variability or change’ (IPCC [61, p 1772]; see also: Smit *et al* [55]), resulting in reduced adaptive capacity. That warming trends differ by level of social vulnerability implies spatially varying inequities in climate change impacts. However, this disparity may be somewhat narrowed by the opposite relationship with warm-tail variability, since the most-vulnerable counties have seen an increased spread of *HI*_*max*_ while the least vulnerable have not, on average. Nonetheless, we can observe that some locations—such as the Mississippi Alluvial Plain—not only have relatively high rates of *HI*_*max*_ warming by median and 95th percentile, but also have both increasing warm-tail spread and amongst the highest values of the Social Vulnerability Index nationally. Analyzing warm-season heat changes through this lens of climatic sensitivity may have utility both in identifying the communities most severely affected and in determining how best to facilitate effective and equitable adaptation.

The interaction between these components—differential warming, changes in heat variability, and higher social vulnerability—may have its most direct implications on public health. There is existing evidence of heat-health mortality carrying greater risks for socially vulnerable sub-populations such as the elderly, young, and those with pre-existing medical conditions (e.g., Reid *et al* [3] and Reid *et al* [62]), so it would be expected that, all else constant, heat mortality would be greatest in places with the highest social vulnerability. However, many other factors influence the epidemiologic impact of heat, including the aforementioned acclimatization that renders cooler climates more sensitive to extreme heat, on average[60, 63]. While others have noted that the rate of reductions in overall heat-related deaths during the past several decades [4, 64, 65] has likely exceeded the rate of mean temperature changes [66], there is a dearth of literature isolating the role of climate change directly in these temporal epidemiologic trends [67]. The recognition that higher statistical moments, such as within-season variability, have discrete health effects [11, 12] suggests the need for more-nuanced analyses of climate-change induced effects on heat mortality, considering not only changes in mean states, but also in the extremes and within-season variability. It is plausible, for example, that the rate of decline in heat-related deaths was less steep in places with greater warming and greater variability, such as in the Northeast or Midwest. Future work should therefore attempt to disentangle the complicated interplay between specific climatic changes and social vulnerability to identify the most effective adaptive interventions.

## 5. Conclusion

The findings presented here suggest regional heterogeneity across CONUS in recent trends (1979–2018) of median and 95th percentiles of maximum daily heat index (*HI*_*max*_) during the warm season (May-September [MJJAS]), with particularly strong warming across the eastern half of the country. Although CONUS overall appears to have experienced substantial warming of *HI*_*max*_ (i.e., rightward shift of the distribution), our data show only a very small increase in intra-seasonal warm-tail *spread* of *HI*_*max*_ (as measured by the trend in the seasonal difference between the 95th and 50th percentiles) nationally. However, the sign and magnitude of warm-tail spread differs strongly between regions: the Midwest and Northeast show positive trends, apparently driven by the faster rate of change in extreme (relative to median) *HI*_*max*_, while the western US, Northern Great Plains, and Southeast show the opposite relationship. The most-vulnerable decile of counties, on average, appears to be warming at a faster rate than the least-vulnerable decile of counties at both the median and 95th percentile, suggesting an inequitable distribution of climatic sensitivity. However, we did not observe the same relationship in the warm-tail spread, since the most-vulnerable counties were seen to have virtually no change while the least-vulnerable counties have trended toward increased spread. Future inquiries are needed not only to determine the geophysical drivers of these spatially differential changes in *HI*_*max*_ distributions, but also to discern the net impact on heat mortality from covarying changes in social vulnerability, atmospheric warming, and distributional variability.
